# Prevalence and associated factors of adolescent tobacco use in Kingdom of Saudi Arabia: Findings from nationwide survey

**DOI:** 10.1371/journal.pgph.0006434

**Published:** 2026-05-04

**Authors:** Ashfaq Ahmad Shah Bukhari, Yasmin Alaa El-din Ali Youssef, B K Manjunatha Goud, Naseer K. Almaliky, Isra’a M. Essa, Taghreed Mohammed Alghaith, Mukhammadali Buriyev, Doniyor Umarov, Mukhayya Djumaniyazova, Majid Mqbel Alkhalaf, Abdullah Nasser Almulhim, Mohammed Saleh Albeshri, Shaima Baker Alsultan, Yousef Abdullah Albalawi, Rham Abdulhadi Alamri, Abdulilah Alqarny, Mouhammed Sleiay, Mahmoud M. Ali

**Affiliations:** 1 Department of Physiology, RAK College of Medical Sciences, Ras Al Khaimah Medical and Health Sciences University, Ras Al Khaimah, United Arab Emirates; 2 Department of Sociology, Faculty of Women for Arts, Science and Education, Ain-Shams University, Cairo, Egypt; 3 Department of Biochemistry, RAKCOMS, RAK Medical and Health Sciences University, Ras Al Khaimah, United Arab Emirates; 4 Department of Surgery, College of Medicine, University of Wasit, Wasit, Iraq; 5 Department of Public Health, College of Veterinary Medicine, University of Basrah, Basra, Iraq; 6 Public Health Authority, Riyadh, Saudi Arabia; 7 Department of Medicine, Termez University of Economics and Service, Termez, Uzbekistan; 8 Department of Clinical Sciences, Ma’mun University, Urgench, Uzbekistan; 9 Department of Pedagogy and Psychology, Urganch State University, Urgench, Uzbekistan; 10 Ministry of Health, Madinah, Saudi Arabia; 11 Ministry of Health, Jazan, Kingdom of Saudi Arabia; 12 Faculty of Medicine, Hama University, Hama, Syria; 13 Nabarouh Health Affairs Directorate, Ministry of Health and Population, Dakahlia Governorate, Nabarouh, Egypt; The Chinese University of Hong Kong Faculty of Medicine, HONG KONG

## Abstract

Tobacco use remains a critical public health issue in Saudi Arabia, where tobacco-related illnesses are a leading cause of preventable mortality. This study assessed the prevalence and associated factors of tobacco use among Saudi adolescents using data from the 2022 Global Youth Tobacco Survey (GYTS). A total of 5,610 students aged 13–15 years were analyzed. The overall tobacco use prevalence was 12.8% (n = 682), with higher rates among older adolescents and males. The main adjusted factors associated with current tobacco use were: male sex (adjusted OR = 1.531, 95% CI: 1.298–1.806, p < 0.001); having close friends who smoke tobacco, compared with none of them smoking (some of them: adjusted OR = 3.553, 95% CI: 2.892–4.363, p < 0.001; most of them: adjusted OR = 3.740, 95% CI: 2.597–5.386, p < 0.001; all of them: adjusted OR = 12.235, 95% CI: 6.951–21.534, p < 0.001); and perceiving tobacco use as “more attractive” (adjusted OR = 1.399, 95% CI: 1.090–1.795, p = 0.008), whereas perceiving tobacco use as “less attractive” was associated with lower odds (adjusted OR = 0.686, 95% CI: 0.563–0.836, p < 0.001). These findings highlight the need for targeted interventions such as school-based prevention programs, parental education initiatives, peer-led anti-tobacco activities, and campaigns by health authorities, including the Public Health Authority (Weqaya), to correct misconceptions about the social appeal of tobacco use and strengthen tobacco control efforts among youth in Saudi Arabia.

## Introduction

Tobacco use remains a major global public health challenge and the leading preventable cause of morbidity and premature mortality [1]. Non-communicable diseases (NCDs), which account for most tobacco-related deaths, are the leading cause of adult mortality in the Kingdom of Saudi Arabia (KSA), responsible for 78% of all deaths [2]. Smoking-related diseases contribute significantly to this burden, with an estimated 70,000 deaths annually in KSA [3]. Although tobacco use prevalence has declined in some settings over time, KSA continues to face a substantial tobacco-use burden [4,5].

In 2022, the adult current cigarette-smoking prevalence in KSA was 16.2%, rising from 12.7% in 2019, while estimates of any tobacco product use among adults reached approximately 17.8% in 2022 [6]. Among adult tobacco users in KSA, cigarettes were the most reported tobacco product, followed by waterpipe (shisha) use and vaping; however, these percentages may not be mutually exclusive because some individuals may use more than one product type [6]. Despite some progress, adolescents’ tobacco use remains a pressing concern [7]. World Health Organization (WHO) data from 2016 reported that 21.2% of male and 9.1% of female adolescents aged 13–15 years in KSA were current tobacco users. The 2022 GYTS assessed tobacco use in the same age group, allowing clearer interpretation of changes over time [8].

More recent findings from national surveys according to Global Youth Tobacco Survey (GYTS) indicate that in 2022, 9.4% of children and adolescents were current tobacco users, with a higher prevalence among boys (10.6%) than girls (8%) [6]. In KSA the economic burden of tobacco use remains high. According to the Tobacco Atlas, the annual cost of tobacco-use in KSA has been estimated at 38,847,780,689 Saudi Riyals (≈US $10.36 billion), which constitutes the largest share among Gulf Cooperation Council [9]. In the KSA context, several cultural and social dynamics warrant special attention to youth tobacco use [10].

Despite the implementation of tobacco control policies aligned with World Health Organization Framework Convention on Tobacco Control WHO guidelines, KSA faces persistent challenges in enforcement, particularly regarding sales to minors and the proliferation of single-stick cigarette sales [11]. Additionally, the social acceptability of male smoking, peer group dynamics, and shifting social norms due to globalization and digital media exposure create a unique environment that fosters adolescent tobacco experimentation [12].

Use of emerging tobacco and nicotine products, particularly vaping devices and heated tobacco products, has become an important consideration in youth tobacco surveillance in Saudi Arabia. Because the 2022 Saudi GYTS captured product-specific measures relevant to adolescent tobacco use, including vaping and heated tobacco products, these products provide important context for interpreting contemporary youth tobacco use patterns in KSA [13]. Furthermore, cultural factors such as familial influence, especially maternal tobacco use which is traditionally stigmatized but on the rise may alter youth tobacco use patterns in ways not fully understood in prior research [14].

A gap persists between research evidence and youth tobacco control policies in the KSA. Although data on adolescent tobacco use exist, they are not always translated into targeted prevention strategies addressing key drivers. Therefore, this study used the 2022 Global Youth Tobacco Survey to update prevalence estimates and identify associated factors to support evidence-based policy and prevention efforts.

## Methods

In 2022, the Saudi Ministries of Health and Education conducted a nationally representative, school-based cross-sectional study using the GYTS to assess tobacco use among students aged 13–15 (grades 1–3 of intermediate school). This secondary analysis draws on that dataset to examine tobacco use prevalence and associated factors among intermediate students aged 13–15 across KSA.

### Sampling

The data for this study were obtained from the Saudi Arabian implementation of the GYTS, conducted between October 1, 2022, and December 15, 2022. The GYTS is a standardized, school-based, cross-sectional survey developed by the WHO and the U.S. Centers for Disease Control and Prevention (CDC) to monitor youth tobacco use and tobacco control indicators globally.

The specific timing of the Saudi GYTS ensures that the findings are contemporaneous with the current public health and tobacco use landscape in KSA. Reporting the exact survey period improves the contextualization of the findings and supports reproducibility. GYTS uses a globally standardized, two-stage cluster sampling design, in which schools are selected with probability proportional to enrollment size at the first stage and classes are randomly selected at the second stage, with all students in selected classes eligible to participate [6]. For the Saudi GYTS, the resulting national dataset was analyzed using the standard GYTS sampling weights for descriptive estimates.

GYTS utilizes a standard core questionnaire, supplemented by optional country specific questions, to measure key tobacco control indicators. The questionnaire assesses tobacco use (smoked and smokeless), cessation, secondhand smoke exposure, tobacco advertising and promotion, product access, and tobacco-related knowledge and attitudes. Participants complete self-administered, anonymous questionnaires via scannable bubble sheets, ensuring confidentiality [6].

### Sample size and missing data

A total of 6,983 students aged 13–15 years were eligible to participate in the 2022 Saudi GYTS. After excluding observations with missing values on key variables required for the main descriptive analysis (age, current tobacco use status, parental tobacco use, peer tobacco use, and weekly spending money), the final analytic sample comprised 5,610 adolescents. Overall missing data accounted for less than 6% of the eligible sample. We examined the distribution of missingness across age, sex, and school grade and found no indication of systematic differences, supporting the assumption that the data were approximately missing completely at random (MCAR), although MCAR cannot be formally verified from the available data alone [15]. Descriptive prevalence estimates were calculated using this final analytic sample of 5,610 adolescents. For the logistic regression analyses, we used pairwise deletion, which retains all available observations for each model; therefore, the effective denominator could vary slightly across regression models depending on missingness in the included covariate. This approach is considered acceptable under low missingness and an MCAR mechanism, as the literature indicates consistent estimates for pairwise deletion when the MCAR assumption holds [15].

### Definition of tobacco use

Tobacco use in this study was measured in accordance with the GYTS standard definitions to ensure comparability with global and regional data. Current tobacco use was operationalized as having tobacco use on at least one day during the past 30 days, consistent with WHO guidelines and previous studies on adolescent tobacco use. This definition captures both regular and occasional current tobacco user, providing a comprehensive measure of active smoking behavior among youth. Additionally, the survey assessed the use of electronic nicotine delivery systems (vaping) and waterpipe (shisha) smoking, also based on past 30-day use. These clear operational definitions facilitate accurate measurement, align with global surveillance standards, and ensure consistency with prior research conducted in KSA and internationally.

### National representativeness of the sample

The sample for this study is nationally representative of adolescents aged 13–15 years across KSA. The 2022 Saudi GYTS employed a two-stage cluster sampling design. In the first stage, schools were selected proportionally based on enrollment size. In the second stage, classes within selected schools were randomly sampled, and all students in those classes were invited to participate. This methodological approach ensures that the findings can be generalized to the broader adolescent population within KSA, covering all administrative regions.

### Covariate selection and rationale for exclusion

In the present analysis, the selection of covariates was informed by the variables available in the 2022 Saudi GYTS dataset and by prior literature on adolescent tobacco use. The multivariable models included age, sex, school level, peer tobacco use, parental tobacco use, weekly spending money, and perceived attractiveness of tobacco use. These factors are among the most proximal and modifiable associated factors of youth tobacco use behavior. Although covariates such as parental education, household income, and urban versus rural residence are commonly included in tobacco use research, these variables were not collected in the 2022 Saudi GYTS dataset. As such, they were not available for inclusion in the statistical models. The absence of these broader socioeconomic and contextual variables means that some residual confounding is possible, particularly for associations involving school level, weekly spending money, and parental tobacco use, which may partially reflect underlying family socioeconomic position or neighborhood characteristics. Consequently, the estimated effects of the included covariates should be interpreted as adjusted for the available proximal behavioral and demographic factors, but not fully adjusted for all structural social determinants. This limitation is acknowledged and reflects the constraints of secondary data analysis. Nevertheless, the covariates used in this study capture critical social, demographic, and behavioral determinants that are directly relevant to adolescent tobacco use initiation in the Saudi context.

### Ethics statement

The research was conducted using secondary data from the 2022 SGYTS. Ethical approval and data access permissions were obtained from the responsible authorities (NCD Surveillance, Monitoring and Reporting Team - World Health Organization). The study adhered to the ethical standards outlined in the Declaration of Helsinki (1964 and its amendments in 2008). Since the analysis involved anonymized public health surveillance data, individual informed consent was not required beyond the GYTS protocols.

### Statistical analysis

All statistical analyses were conducted utilizing SPSS software, specifically Version 27. Descriptive statistics were employed to ascertain the prevalence of tobacco use among the participants. To assess differences in tobacco use status based on various sociodemographic characteristics of adolescents, the chi-square test was utilized. Univariate logistic regression analyses were first performed to estimate crude odds ratios (ORs) and 95% confidence intervals (CIs) for the association between current tobacco use and each covariate of interest. A single multivariable logistic regression model was then fitted for current tobacco use, including the pre-specified covariates age, sex, school level, peer tobacco use, parental tobacco use, weekly spending money, and perceived attractiveness of tobacco use. Covariate inclusion was determined a priori based on prior literature, conceptual relevance, and availability in the 2022 Saudi GYTS dataset, rather than by stepwise variable selection. The Cox and Snell R^2^ was calculated to assess the goodness-of-fit of the logistic regression models. All descriptive statistics, including prevalence estimates, were weighted using the standard GYTS sampling weights to adjust for the complex survey design, non-response, and post-stratification by sex and grade. This ensures national representativeness of the findings. All monetary values in the dataset, including weekly spending categories, are reported in Saudi Arabian Riyals (SAR), the national currency of KSA.

## Results

From a total of 6,983 eligible intermediate school students surveyed, 5,610 aged 13–15 years were enrolled, yielding an overall response rate of 92.3%. Overall, 31.1% of adolescents reported lifetime tobacco use, while 12.8% were classified as current tobacco users.

Tobacco use prevalence increased significantly with age, being 9.1% among 13 year-olds, 13.0% among 14-year-olds, and 17.1% among 15-year-olds (P < 0.001). Males had a higher prevalence than females (15.0% vs. 10.4%, P < 0.001). Tobacco use also varied by school level, with rates of 9.6% in 1st intermediate, 13.1% in 2nd, and 15.4% in 3rd intermediate students (P < 0.001). Weekly spending was significantly associated with tobacco use (P = 0.007), with the highest prevalence among those spending ≥100 SAR (15.3%) and the lowest among those spending less than 30 SAR (10.5%) (Table 1).

**Table 1:** Current prevalence of adolescents’ tobacco use by sociodemographic characteristics.

Characteristics	Current tobacco user No.	Current tobacco user percentages*	Non-users No.	Non-users Percentages*	P-value
Age Group in years	682		4640		<0.001**
13	165	9.1%	1653	90.9%	
14	267	13.0%	1779	87.0%	
15	250	17.1%	1208	82.9%	
Sex	676		4612		<0.001**
Female	264	10.4%	2284	89.6%	
Male	412	15.0%	2328	85.0%	
School Level	673		4602		<0.001**
1st Intermediate	151	9.6%	1420	90.4%	
2nd Intermediate	280	13.1%	1853	86.9%	
3rd Intermediate	242	15.4%	1329	84.6%	
Weekly spending money	665		4609		0.007**
I usually do not have any spending money	156	13.9%	1019	86.1%	
Less than 30 SAR	196	10.5%	1669	89.5%	
30–49 SAR	143	13.4%	923	86.6%	
50–99 SAR	98	14.1%	599	85.9%	
100 SAR or more	72	15.3%	399	84.7%	

Percentages are rounded. **Significant. 1 USD = 3.75 SAR.

In the adjusted analysis, compared with 13-year-olds, adolescents aged 14 years and 15 years had higher odds of current tobacco use (aOR = 1.469, 95% CI: 1.192–1.810, P < 0.001; and aOR = 2.001, 95% CI: 1.609–2.488, P < 0.001, respectively). Male adolescents also had higher odds of current tobacco use than females (aOR = 1.523, 95% CI: 1.286–1.805, P < 0.001). Relative to students in the 1st intermediate level, those in the 2nd and 3rd intermediate levels had higher adjusted odds of current tobacco use (aOR = 1.378, 95% CI: 1.115–1.703, P = 0.003; and aOR = 1.647, 95% CI: 1.318–2.058, P < 0.001, respectively). Adolescents spending less than 30 SAR weekly had lower adjusted odds of tobacco use (aOR = 0.720, 95% CI: 0.575–0.901, P = 0.004), whereas the remaining spending categories were not significantly associated with tobacco use after adjustment (Table 2).

**Table 2:** Crude and adjusted associations of sociodemographic factors with adolescents’ tobacco use.

Characteristics	Current tobacco user	Non-users	OR for crude estimates	95% CI	P-value for OR for crude estimates	OR adjusted*	95% CI	P-value for adjusted OR
Age in years (Ref: 13)								
14	267	1779	1.504	1.225–1.846	<0.001	1.469	1.192–1.810	<0.001
15	250	1208	2.073	1.680–2.558	<0.001	2.001	1.609–2.488	<0.001
Sex (Ref: Female)								
Male	412	2328	1.531	1.298–1.806	<0.001	1.523	1.286–1.805	<0.001
School level (Ref: 1st Intermediate)								
2nd Intermediate	280	1853	1.421	1.152–1.752	0.001	1.378	1.115–1.703	0.003
3rd Intermediate	242	1329	1.712	1.379–2.126	<0.001	1.647	1.318–2.058	<0.001
Weekly spending money (Ref: I usually do not have any spending money)								
Less than 30 SAR	196	1669	0.725	0.581–0.905	0.004	0.720	0.575–0.901	0.004
30–49 SAR	143	923	1.009	0.787–1.293	0.942	0.936	0.733–1.197	0.600
50–99 SAR	98	599	1.067	0.803–1.416	0.656	1.001	0.761–1.318	0.993
100 SAR or more	72	399	1.176	0.851–1.625	0.324	1.094	0.803–1.491	0.568

Descriptive prevalence estimates were weighted using standard GYTS sampling weights. Logistic regression odds ratios are presented as model-based estimates; confidence intervals and p-values may not fully account for clustering and stratification in the complex survey design. OR = Odds Ratio CI = Confidence Interval Ref. = Reference category. 1 USD = 3.75 SAR. * OR adjusted for other variables in this table.

Similarly, in the adjusted analysis of smoking-related factors, parental tobacco use remained significantly associated with current tobacco use. Compared with adolescents whose parents did not smoke, the adjusted odds of tobacco use were higher among those reporting that both parents smoked (aOR = 2.800, 95% CI: 1.954–4.011, P < 0.001), father only smoked (aOR = 1.922, 95% CI: 1.533–2.410, P < 0.001), mother only smoked (aOR = 4.919, 95% CI: 2.450–9.878, P < 0.001), or who did not know their parents’ smoking status (aOR = 1.917, 95% CI: 1.335–2.911, P < 0.001). Peer influence showed the strongest association, with higher adjusted odds of tobacco use among adolescents whose closest friends smoked tobacco, compared with none of them smoking (some of them: aOR = 3.553, 95% CI: 2.892–4.363, P < 0.001; most of them: aOR = 3.740, 95% CI: 2.597–5.386, P < 0.001; all of them: aOR = 12.235, 95% CI: 6.951–21.534, P < 0.001). In addition, perceiving tobacco use as more attractive was associated with higher adjusted odds of current tobacco use (aOR = 1.399, 95% CI: 1.090–1.795, P = 0.008), whereas perceiving it as less attractive was associated with lower adjusted odds (aOR = 0.686, 95% CI: 0.563–0.836, P < 0.001) (Table 3).

**Table 3:** Crude and adjusted associations of smoking-related factors with adolescents’ tobacco use.

Characteristics	Current tobacco user	Non-users	OR for crude estimates	95% CI	P-value for OR for crude estimates	OR adjusted*	95% CI	P-value for adjusted OR
Parents smoke tobacco (Ref: None of them)								
Both	46	134	2.820	1.990–3.996	<0.001	2.800	1.954–4.011	<0.001
Father only	119	512	1.909	1.530–2.383	<0.001	1.922	1.533–2.410	<0.001
Mother only	13	23	4.644	2.336–9.229	<0.001	4.919	2.450–9.878	<0.001
Don’t know	34	149	1.875	1.276–2.754	0.001	1.917	1.335–2.911	<0.001
Closest friends smoke tobacco (Ref: None of them)								
Some of them	177	441	3.849	3.148–4.707	<0.001	3.553	2.892–4.363	<0.001
Most of them	45	111	3.888	2.711–5.576	<0.001	3.740	2.597–5.386	<0.001
All of them	31	22	13.513	7.754–23.551	<0.001	12.235	6.951–21.534	<0.001
Perceived attractiveness of smoking (Ref: No difference)								
More attractive	143	565	1.455	1.141–1.857	0.003	1.399	1.090–1.795	0.008
Less attractive	353	3016	0.673	0.554–0.817	<0.001	0.686	0.563–0.836	<0.001

OR = Odds Ratio CI = Confidence Interval Ref. = Reference category. 1 USD = 3.75 SAR. * OR adjusted for other variables in this table.

The regression model identified peer tobacco use as the strongest factor associated with adolescent tobacco use, with odds ratios of 3.553 (95% CI: 2.892–4.363; P < 0.001) for having some smoking friends, 3.740 (95% CI: 2.597–5.386; P < 0.001) for having most smoking friends, and 12.235 (95% CI: 6.951–21.534; P < 0.001) for having all smoking friends, compared to none. Parental tobacco use was also significantly associated with higher odds of tobacco use: OR = 2.800 (95% CI: 1.954–4.011; P < 0.001) for both parents, OR = 1.922 (95% CI: 1.533–2.410; P < 0.001) for father only, OR = 4.919 (95% CI: 2.450 9.878; P < 0.001) for mother only, and OR = 1.917 (95% CI: 1.335–2.911; P < 0.001) for those who did not know the parental status, compared to those whose parents did not smoke. Spending less than 30 SAR weekly was associated with reduced odds (OR = 0.720; 95% CI: 0.575–0.901; P = 0.004), while other spending levels showed no significant associations. Believing that tobacco use makes youth more attractive was associated with higher odds of tobacco use (OR = 1.399; 95% CI: 1.090–1.795; P = 0.008), whereas seeing it as less attractive was associated with lower odds (OR = 0.686; 95% CI: 0.563–0.836; P < 0.001) (Table 4).

**Table 4:** Primary multivariable model of factors associated with adolescents’ tobacco use.

Characteristics	Categories	Current tobacco user	Non-users	OR adjusted*	95% CI	P-value
Parents smoke tobacco	None of them	464	3812	Ref	Ref	
	Both	46	134	2.800	1.954–4.011	<0.001
	Father only	119	512	1.922	1.533–2.410	<0.001
	Mother only	13	23	4.919	2.450–9.878	<0.001
	Don’t know	34	149	1.917	1.335–2.911	<0.001
Closest friends smoke tobacco	None of them	422	4047	Ref	Ref	
	Some of them	177	441	3.553	2.892–4.363	<0.001
	Most of them	45	111	3.740	2.597–5.386	<0.001
	All of them	31	22	12.235	6.951–21.534	<0.001
Weekly spending money	I usually do not have any spending money	156	1019	Ref	Ref	
	Less than 30 SAR	196	1669	0.720	0.575–0.901	0.004
	30–49 SAR	143	923	0.936	0.733–1.197	0.600
	50–99 SAR	98	599	1.001	0.761–1.318	0.993
	100 SAR or more	72	399	1.094	0.803–1.491	0.568
Perceived attractiveness of smoking	No difference	176	1012	Ref	Ref	
	More attractive	143	565	1.399	1.090–1.795	0.008
	Less attractive	353	3016	0.686	0.563–0.836	<0.001

Descriptive prevalence estimates were weighted using standard GYTS sampling weights. Logistic regression odds ratios are presented as model-based estimates; confidence intervals and p-values may not fully account for clustering and stratification in the complex survey design * OR adjusted for age, sex, and school level. Standard abbreviations: OR = Odds Ratio; CI = Confidence Interval; Ref. = Reference category. 1 USD = 3.75 SAR.

Use of more than one tobacco product was significantly associated with age, sex, and school grade. Older students showed higher odds of using more than one tobacco product compared with 13-year-olds, with the strongest increase observed at age 15 (OR = 1.897; 95% CI: 1.541 2.336; P < 0.001). Fourteen-year-olds also showed increased odds (OR = 1.232; 95% CI: 1.002–1.516; P = 0.048). Males had higher odds than females (OR = 1.230; 95% CI: 1.042 1.451; P = 0.014), and students in the 3rd intermediate grade demonstrated increased odds relative to 1st-grade students (OR = 1.783; 95% CI: 1.441–2.207; P < 0.001). Weekly spending money was not significantly associated with multiple tobacco product use (Table 5).

**Table 5:** Sociodemographic factors associated with using more than one tobacco product among adolescents in the analytic sample.

Characteristics	Multiple tobacco use (Yes)	Multiple tobacco use (No)	OR for crude estimates	95% CI	P-value for OR for crude estimates	OR adjusted*	95% CI	P-value for adjusted OR
Age in years (Ref: 13)								
14	154	1892	1.232	1.002–1.516	0.048	1.159	0.909-1.478	0.235
15	207	1251	1.897	1.541–2.336	<0.001	1.539	1.146-2.066	0.004
Sex (Ref: Female)								
Male	238	2502	1.230	1.042–1.451	0.014	1.233	1.041–1.460	0.015
School level (Ref: 1st Intermediate)								
2nd Intermediate	196	1937	1.140	0.929–1.399	0.208	1.013	0.794-1.292	0.916
3rd Intermediate	235	1336	1.783	1.441–2.207	<0.001	1.341	0.994-1.809	0.054
Weekly spending money (Ref: I usually do not have any spending money)								
Less than 30 SAR	235	1630	0.941	0.764–1.158	0.560	0.911	0.724–1.146	0.426
30–49 SAR	149	917	0.941	0.743–1.193	0.615	0.979	0.756–1.267	0.870
50–99 SAR	98	599	1.029	0.776–1.365	0.842	1.243	0.943–1.639	0.123
100 SAR or more	72	399	1.127	0.824–1.540	0.454	1.185	0.861–1.631	0.298

OR = Odds Ratio; CI = Confidence Interval; Ref. = Reference category.

* OR adjusted for other variables in this table.

Heavier smoking was most concentrated among 15-year-old students, males, 3rd intermediate students, and those with higher weekly spending (50–99 SAR and ≥100 SAR). These groups showed the highest proportions in the upper smoking categories, including 2–5 cigarettes/day (up to 1.2% in 15-year-olds, 1.1% in males, and 1.1% in 3^rd^ intermediate students) and 6–10 cigarettes/day (up to 0.4% in 15-year-olds and 0.3% among those with higher spending) (Table 6).

**Table 6:** Relationship between socioeconomic/demographic characteristics and number of cigarettes smoked per day.

Characteristics	I did not smoke cigarettes during the past 30 days	Less than 1 cigarette per day	1 cigarette per day	2 to 5 cigarettes per day	6 to 10 cigarettes per day	11 to 20 cigarettes per day	20 + cigarettes per day
Age in years							
13	1653(90.52%)	80(4.38%)	37(2.03%)	46(2.52%)	10(0.55%)	0	0
14	1779(87.68%)	127(6.26%)	59(2.91%)	55(2.71%)	9(0.44%)	0	0
15	1208(77.94%)	174(11.23%)	70(4.52%)	74(4.77%)	18(1.16%)	6(0.39%)	0
Sex							
Female	2284(92.02%)	103(4.15%)	46(1.85%)	39(1.57%)	10(0.40%)	0	0
Male	2328(80.41%)	278(9.60%)	120(4.15%)	136(4.70%)	27(0.93%)	6(0.21%)	0
School level							
1st Intermediate	1420(90.39%)	74(4.71%)	30(1.91%)	39(2.48%)	8(0.51%)	0	0
2nd Intermediate	1853(85.67%)	150(6.93%)	70(3.24%)	70(3.24%)	14(0.65%)	6(0.28%)	0
3rd Intermediate	1329(81.43%)	157(9.62%)	65(3.98%)	66(4.04%)	15(0.92%)	0	0
Weekly spending money							
I usually do not have any spending money	1019(85.70%)	85(7.15%)	36(3.03%)	37(3.11%)	12(1.01%)	0	0
Less than 30 SAR	1669(87.70%)	110(5.78%)	52(2.73%)	56(2.94%)	16(0.84%)	0	0
30–49 SAR	923(86.59%)	83(7.79%)	34(3.19%)	26(2.44%)	0	0	0
50–99 SAR	599(86.56%)	57(8.24%)	20(2.89%)	14(2.02%)	2(0.29%)	0	0
100 SAR or more	399(81.93%)	21(4.31%)	12(2.46%)	42(8.62%)	7(1.44%)	6(1.23%)	0

The chi-square test revealed significant associations between age categories and the use of most tobacco products. Shisha smoking (χ^2^ = 21.401; P < 0.001), smokeless tobacco (χ^2^ = 9.519; P = 0.009), vaping (χ^2^ = 36.460; P < 0.001), and conventional cigarettes (χ^2^ = 47.338; P < 0.001) all showed statistically significant relationships with age, while the use of heated tobacco products was not significant (χ^2^ = 1.670; P = 0.434). This indicates that age is a relevant factor in the consumption patterns of most tobacco product types among the studied population (Table 7).

**Table 7:** The association between age categories and using different types of tobacco products.

Tobacco Product	Total Yes	Total No	χ^2^	P-value	Age 13 Yes	Age 13 No	Age 14 Yes	Age 14 No	Age 15 Yes	Age 15 No
Shisha smoking	824	4629	21.401	<0.001	237	1626	308	1768	279	1235
Smokeless tobacco products	481	4799	9.519	0.009	158	1654	162	1847	161	1298
Vaping use	779	4676	36.460	<0.001	223	1650	272	1802	284	1224
Heated tobacco products	408	5029	1.670	0.434	147	1721	143	1925	118	1383
Cigarettes smoking	682	4640	47.338	<0.001	165	1653	267	1779	250	1208

## Discussion

This national study, using the 2022 GYTS, examined the prevalence and factors associated with tobacco use among Saudi Arabian adolescents. The analysis showed that 31.1% of adolescents reported lifetime tobacco use, while 12.8% were classified as current tobacco users. In the adjusted analyses, older age, male sex, higher school level, parental tobacco use, peer tobacco use, and perceiving tobacco use as more attractive were associated with higher odds of current tobacco use, whereas lower weekly spending money was associated with lower odds. Among these factors, peer tobacco use showed the strongest association with adolescent tobacco use.

Our estimated prevalence of tobacco use is broadly consistent with earlier studies. For instance, a systematic review highlighted that tobacco use prevalence among Saudi adolescents varied significantly, ranging from 9.72% to 37% among secondary school students, 2.4% to 30.9% among college students, and 12.7% to 39.6% among adolescents regardless of their educational stages, with a significantly higher prevalence in males compared to females [8]. Another school-based cross-sectional study in Madinah revealed a tobacco use prevalence of 15.17% among adolescents, with significant differences across sociodemographic factors [16]. Another study in the Tabuk region identified that 25.7% of secondary school boys were tobacco users, and 25.9% engaged in shisha smoking [17]. A study in Riyadh reported that 19.5% of students aged 13–15 were current tobacco users, with a higher prevalence among males (31.2%) compared to females (8.9%) [17]. However, these variations may stem from regional differences, cultural norms, and the times of the studies.

Our adjusted analyses identified several sociodemographic factors significantly associated with current tobacco use. Older students, particularly those in higher grades, exhibited a higher likelihood of tobacco use. Our findings are consistent with the notion that risk behaviors such as tobacco use become more common as adolescents grow older [18]. This pattern may be related to several factors. First, older adolescents generally experience greater autonomy and increased freedom, which often leads to experimentation with risk behaviors, including tobacco use [19]. They are more likely to be exposed to peer pressure and social environments where tobacco use is normalized, which may be associated with higher likelihood of initiation [20]. Moreover, as adolescents age, cumulative exposure to pro-smoking cues such as tobacco advertising and social media imagery can reinforce the perception that tobacco use is a normative behavior [21]. In addition, the transition from early to mid-adolescence is often accompanied by heightened stress from academic and social challenges, leading some youth to adopt tobacco use as a coping mechanism [22,23]. Overall, these factors may help explain the higher prevalence of tobacco use observed between ages 13 and 15.

In addition, the adjusted analysis showed that male students had higher odds of current tobacco use than their female counterparts. This gender disparity is consistent with findings from other regions in Saudi Arabia. For example, the Tabuk study reported that 25.7% of male secondary school students were tobacco users [17]. Cultural factors, societal norms, and possibly greater social freedoms for males may contribute to this difference [24]. Furthermore, a systematic review noted that males believe tobacco use could help increase their masculine image and the perception of maturity among peers, while social stigma may be responsible for the lower prevalence of tobacco use among females [8].

The association between higher school grades and increased prevalence of tobacco use suggests that as adolescents progress through their education, they encounter cumulative exposures to risk factors and shifting social dynamics that make tobacco use more likely. In higher grades, students typically experience increased autonomy and less direct parental oversight, providing more opportunities to experiment with behaviors such as tobacco use [19,25,26]. This developmental stage is also marked by stronger peer influences, as older students are more likely to interact with social groups where tobacco use is normalized or even seen as a marker of maturity [27]. Moreover, the school environment itself may change over time, with older students facing academic pressures and social challenges that can lead to stress, prompting some to use tobacco as a coping mechanism [22]. Previous research supports this trend: studies have consistently shown that older adolescents are at greater risk for initiating tobacco use due to cumulative exposure to pro-tobacco use influences ranging from tobacco advertising to peer behaviors and the evolving social dynamics within schools [28,29].

The finding that lower weekly spending (i.e., less than 30 SAR) is associated with reduced odds of tobacco use among adolescents suggests that limited disposable income can serve as a protective factor. Adolescents with less spending money may have fewer opportunities to purchase tobacco products, which could directly limit their experimentation or regular use.

This result aligns with the notion that economic barriers can restrict access to tobacco, even in the presence of other risk factors such as peer influence or parental tobacco use [16]. In contrast, the overall lack of a consistent trend across all spending categories indicates that while financial resources may facilitate access to tobacco, they are not the sole determinant of tobacco use behavior [30]. Other studies conducted in Saudi Arabia have highlighted that social and familial factors often play a more dominant role in influencing adolescent tobacco use than socioeconomic status alone [12]. For instance, peer pressure, exposure to tobacco use behaviors at home, and cultural acceptance may override the influence of disposable income, thereby diluting the direct impact of spending habits on tobacco use rates [31–33].

Moreover, these findings underscore the complexity of socioeconomic influences on health behaviors. Although higher spending might enable adolescents to buy tobacco more easily, it does not necessarily translate into increased tobacco use prevalence if robust social or parental disapproval exists. Conversely, adolescents with limited funds might also experience other risk factors; however, the economic constraint appears to offer some degree of protection in this context. This complexity is mirrored in other studies from the region, where mixed results have been reported regarding the relationship between socioeconomic status and tobacco use [34].

The findings highlight the profound impact of social influences and perceptions on adolescent tobacco use behavior. Notably, parental tobacco use emerged as a significant risk factor, with adolescents whose parents especially when both or the mother smoked being considerably more likely to initiate tobacco use. This observation aligns with previous research in Saudi Arabia, where studies have consistently reported that parental tobacco use serves as a strong model for youth behavior [12,35]. In culturally tight-knit societies such as Saudi Arabia, the influence of parental role modeling is paramount, as it normalizes tobacco use within the home environment and increases the accessibility of tobacco products.

Peer tobacco use showed the strongest association in the multivariable model; adolescents whose all-close friends use tobacco had more than a 12-fold increase in the odds of current tobacco use compared with those whose close friends did not use tobacco. This very high odds ratio is epidemiologically important because it is consistent with the possibility that peer tobacco use may be linked to multiple reinforcing social mechanisms, including social modeling, perceived acceptability, normalization of tobacco use within friendship networks, and easier access to tobacco products through peers[36–44]. The pattern observed across the categories of peer tobacco use (some, most, and all close friends) also suggests a graded association, whereby increasing exposure to tobacco-using peers is associated with progressively higher odds of adolescent tobacco use. Similar findings have been reported in studies conducted among both Saudi and non-Saudi youth, where peer pressure and the desire to fit in with a tobacco use peer group significantly increase the likelihood of tobacco use [12,28,29]. In adolescence, when peer belonging and identity formation are especially influential, having all close friends who use tobacco may create a social environment in which tobacco use is seen as normative, socially rewarding, or difficult to refuse. This may help explain why the magnitude of the association for peer influence exceeded that of most other factors in the present study. Taken together, peer dynamics, alongside parental influence, may be associated with a social environment that is more favorable to the initiation and continuation of tobacco use.

Additionally, the perception that tobacco use enhances attractiveness was associated with increased tobacco use odds. Adolescents who view tobacco as a means to boost their social image may be more likely to use tobacco, which reflects broader societal and media influences that glamorize tobacco use. Such perceptions have been documented, indicating that the social construction of tobacco use as a symbol of maturity or sophistication plays a critical role in its uptake [45]. In Saudi Arabia, where rapid cultural and social changes are influencing traditional norms, these perceptions can be particularly influential.

Together, these findings emphasize that effective tobacco control interventions in Saudi Arabia must address not only the economic and individual risk factors but also the powerful social influences and perceptions that are associated with adolescent tobacco use. Interventions that involve parental education, peer-led initiatives, and media campaigns to counteract the glamorization of tobacco use may prove especially beneficial in mitigating these risks.

## Limitations

The study’s limitations include the use of self-administered questionnaires in the GYTS, which may have led to response bias, and the cross-sectional nature of the data, which prevents causal inference. Accordingly, the observed relationships should be interpreted as associations rather than causal effects, and temporal directionality cannot be established. A longitudinal study is needed to identify associated factors of adolescent tobacco use in KSA. In addition, we recommend conducting qualitative studies in KSA, such as focus group discussions and in-depth interviews with adolescents, parents, and teachers, to explore how parental discussions, school norms, and family tobacco use patterns shape adolescents’ perceptions and decisions about tobacco use.

An important additional methodological limitation is that, although descriptive prevalence estimates were weighted using the standard GYTS sampling weights, the logistic regression analyses did not explicitly account for the full complex survey design of the GYTS, including clustering and stratification. As a result, the reported standard errors, confidence intervals, and p-values from the regression models may be imprecise. Therefore, the regression findings should be interpreted with caution, with greater emphasis on the direction and relative magnitude of the observed associations rather than on strict statistical inference.

Furthermore, although missing data accounted for less than 6% of the eligible sample, the study relied on pairwise deletion in the regression analyses, while the descriptive prevalence analysis was based on a post-exclusion sample of 5,610 adolescents. Therefore, the effective sample size may have varied slightly across regression models depending on the availability of data for the included covariates. The secondary dataset did not include sufficient auxiliary variables to support more robust imputation methods, which represents an additional methodological limitation that should be acknowledged. Also, the absence of data on cumulative tobacco exposure burden (e.g., pack-years) limits the assessment of health risks, and the limited age range suggests inclusion of other adolescent groups in future research.

## Conclusion

Tobacco use among Saudi adolescents was independently associated with age, sex, school level, peer tobacco use, parental tobacco use, perceived attractiveness of tobacco use, and weekly spending money. The strongest adjusted association was observed for peer tobacco use, underscoring the importance of social influence in adolescent tobacco use behavior. These findings support the need for early school-based prevention programs, peer-led anti-tobacco interventions, parental awareness initiatives, and youth-targeted campaigns to counter pro-tobacco use social norms. Future studies are recommended to confirm these findings using longitudinal designs and complex survey regression methods.

## Abbreviation:

GYTS: Global Youth Tobacco Survey
WHO: World Health Organization
CDC: Centers for Disease Control and Prevention
NCDs: Non-Communicable Diseases
SAR: Saudi Arabian Riyal
SPSS: Statistical Package for the Social Sciences
OR: Odds Ratio
CI: Confidence Interval
GCC: Gulf Cooperation Council
